# Utilizing traditional Chinese for the discovery of efficacious new drugs

**DOI:** 10.1186/1479-5876-10-S2-A34

**Published:** 2012-10-17

**Authors:** Andreas Bender

**Affiliations:** 1 University of Cambridge, Cambridge, UK

## 

Western medicines, while being developed using a plethora of powerful research technologies, still often fail in the clinic due to efficacy problems as well as exhibiting side-effects. Part of this problem can be attributed to the development of protein-target centric drug discovery methods, which do not consider the whole organism during the discovery stages, but only a very (possibly over-) simplified model system. Hence, results cannot be translated easily into the clinic, which gives rise to the problems mentioned above.

In this work, we hence propose to utilize the knowledge compiled by traditional medicines (such as Traditional Chinese over large time spans, which consider the phenotypic effect (the effect in man) of treatments from the onset. Recent developments in analytical chemistry enable us to determine the bioactive principles in compound mixtures and *in silico* (computational) protein target prediction tools allow for the speedy determination of target proteins, shortening the time needed for subsequent optimization of formulations. We will focus on diseases where treatments in the West are lacking, such as cardiovascular diseases; cancer; liver fibrosis and liver cirrhosis; as well as diabetic retinopathy, and determine active principle and mode of action of traditional medicines which have shown promise against those diseases.

The particular case studies to be examined initially are Danshen Dripping Pill (angina pectoris and cardiovascular diseases); Ginseng and Ginsenosides (cancer and inflammation); Fu Zheng Hua Yu (liver fibrosis and cirrhosis) and Qiden Mingmu (diabetic retinopathy).

The aim is, at the end of this project, to have bioactive compounds (or mixtures of compounds) at hand which can be further developed into efficacious treatments, with an increased likelihood of success in the clinic.

